# Prolonged Static Whole-Body Roll-Tilt and Optokinetic Stimulation Significantly Bias the Subjective Postural Vertical in Healthy Human Subjects

**DOI:** 10.3389/fneur.2020.595975

**Published:** 2020-10-15

**Authors:** Andrea Wedtgrube, Christopher J. Bockisch, Dominik Straumann, Alexander A. Tarnutzer

**Affiliations:** ^1^Department of Neurology, University Hospital Zurich, Zurich, Switzerland; ^2^Department of Otorhinolaryngology, University Hospital Zurich, Zurich, Switzerland; ^3^Department of Ophthalmology, University Hospital Zurich, Zurich, Switzerland; ^4^Faculty of Medicine, University of Zurich, Zurich, Switzerland; ^5^Center of Clinical Neurosciences, University Hospital Zurich, Zurich, Switzerland; ^6^Neurology, Cantonal Hospital of Baden, Baden, Switzerland

**Keywords:** graviception, vestibular, Bayesian modeling, adaptation, postural vertical

## Abstract

**Background:** Prolonged static whole-body roll-tilt has been shown to bias estimates of the direction of gravity when assessed by static paradigms such as the subjective visual vertical and the subjective haptic vertical.

**Objective:** We hypothesized that these shifts are paradigm-independent and thus predicted a post-tilt bias as well for self-adjustments along perceived vertical (subjective postural vertical, SPV). Likewise, rotatory optokinetic stimuli, which have been shown to shift the SPV when presented at the time of adjustments, may have an lasting effect on the SPV, predicting a shift in the perceived direction of gravity in the direction of the optokinetic rotatory stimulation.

**Methods:** Self-adjustments along perceived vertical by use of a motorized turntable were recorded at baseline and after 5 min of static whole-body roll-tilt (orientation = ±90°, adaptation period) in 10 healthy human subjects. During adaptation subjects were either in darkness (no OKN stimulation) or were presented a full-field rotatory optokinetic stimulus (velocity = ±60°/s). Statistical analysis of adjustment errors for the different conditions was performed using a generalized linear model.

**Results:** After 5 min of static whole-body roll-tilt in darkness, we observed significant (*p* < 0.001) shifts in the SPV averaging −2.8° (adaptation position: −90°) and 3.1° (+90°), respectively. Adding an optokinetic rotatory stimulus resulted in an additional, significant shift of SPV adjustments toward the direction of the previously presented optokinetic rotation (optokinetic clockwise rotation: 1.4°, *p* = 0.034; optokinetic counter-clockwise rotation: −1.3°, *p* = 0.037). Trial-to-trial variability of turntable adjustments was not significantly affected by adaptation.

**Conclusions:** Prolonged static roll-tilt results in a significant post-tilt bias of the perceived direction of gravity when assessed by the SPV, confirming previous findings from other vision-dependent and vision-independent paradigms. This finding emphasizes the impact of recent whole-body roll orientations relative to gravity. Such adaptational shifts in verticality estimates may be explained in the context of Bayesian optimal observer theory with a bias of prior knowledge (i.e., expectation biased by experience). Our findings also have clinical implications, as the observed post-tilt bias may contribute to postural instability when standing up in the morning with an increasing risk for falls and fall-related injuries in humans with preexisting balance disorders.

## Introduction

Human spatial orientation and navigation combines and weights sensory input from different end organs, including the vestibular organs [semicircular canals (SCCs) and otolith organs], pressure sensors in the skin and the visual system (1). For verticality perception, accurate, and precise adjustments have been shown for whole-body positions near upright, whereas for roll-tilted positions systematic roll over- and underestimation has been demonstrated for vision-dependent paradigms such as the subjective visual vertical (SVV) (2–4), but not for vision-independent paradigms such as the subjective haptic vertical (SHV) (5) or the subjective haptic horizontal (6). These differences emphasize the role of central integration of sensory input and also point to resulting biases.

Perceived direction of vertical also depends on the subject's recent history. Specifically, when returning back upright after prolonged static roll-tilt, a systematic bias (termed *post-tilt bias*) in the SVV can be seen (2, 7–10). This bias has exponential decay characteristics (10). It has been postulated that the sensory stimulation during the prolonged roll-tilt shifts the expectation of the body roll position toward the roll-tilt position, and this prior expectation biases perception when upright (10).

Previously, we have shown a similar pattern using a vision-independent paradigm (i.e., the SHV), proposing that this post-tilt bias most likely is of central origin (consistent with the shifting null hypothesis) (11). In most studies [including ours (10, 11)], subjects were passively brought into the roll-tilted adaptation position and back upright afterwards again. In daily life, however, self-positioning along perceived vertical is repeatedly required, e.g., when standing up in the morning after a night's sleep. Such an active task will integrate SCC input and thus differs from the SVV and the SHV task. Nevertheless, otolith input will be available for all these different tasks and thus likely will be integrated as well (12). Since the otolith organs are the only sensors that directly sense the pull of gravity (13), they are considered essential for verticality perception in all these tasks and thus may play a central role in adaptational effects in both active self-positioning in space and paradigms collected while remaining in a static whole-body roll-tilted position such as the SHV or the SVV. Thus, we predict a similar post-tilt bias when subjects are asked to align themselves along the perceived direction of vertical, a task referred to as the “subjective postural vertical” (SPV) [see (14) for review]. In addition to the proposed modulatory effect of prolonged whole-body static roll-tilt on the SPV, we hypothesized that task performance could be additionally biased by optokinetic rotatory stimuli. When presented during the SVV task, this results in a significant, roll-tilt dependent bias of perceived vertical (15), whereas only minor shifts can be seen when using a vision-independent paradigm (16). This prompted us to use this stimulus for adaptational purposes during prolonged static roll-tilt, postulating an additional modulatory effect on verticality perception when back upright. Noteworthy, for the SVV and the SHV we did not observe such a modulatory effect of optokinetic stimulation on perceived vertical when assessed immediately after returning back upright (11). This was possibly related to the fact that the SVV and the SHV task were performed in static positions, thus any adaptational effects on the percept of whole-body rotations may not have had any impact on these paradigms. In contrast, for the self-adjustments in the SPV task SCC input is also available. Hence, we predicted a modulatory effect on human self-positioning in space performance both by prolonged static roll-tilt and by rotatory optokinetic stimuli.

## Materials and Methods

### Study Subjects and Ethics Statement

Six males and four females (aged between 23 and 42 years) completed the SPV paradigm and were included in the study. All participating subjects agreed to and signed a written informed consent, obtained after a meticulous explanation of the experimental procedure. The local ethics committee (Cantonal Ethics Committee Zurich, BASEC 2016-00023) approved the experimental protocol. The protocol was in accordance with the ethical standards of the 2013 Declaration of Helsinki for research involving human beings.

### Experimental Setup

All data was collected on a three-axis motor-driven turntable (prototype built by Acutronic, Jona, Switzerland) and the participants were secured on the turntable with a four-point safety belt. The head was restrained using a thermoplastic mask (Sinmed, Reeuwyk, The Netherlands) which covered most of the head, thus allowing a natural straight-ahead position. The mask supported the wearing of glasses, if needed. Pillows were placed in the gaps the sides of the chair and body regions prone to unwanted movements (i.e., the shoulders, hips, and legs).

The most important organs for graviception are the otolith organs, which are located in the head. Therefore, the subjects' orientation in the roll plane will be referred as head-roll orientation, even though roll movements of the turntable were whole body. The roll axis of the motorized turntable corresponded to the naso-occipital line passing between the subject's eyes.

The optokinetic rotatory stimulus was projected onto a sphere placed 1.5 m in front of the subject by means of a turntable-fixed video projector. The rotating optokinetic stimulus was generated with the Psychophysics Toolbox (17, 18) and GNU Octave (version 3.2.3), and consisted of randomly placed white dots on a black background (15). Three different visual-stimulus trial conditions were applied: baseline (no optokinetic stimulation), a clockwise rotating optokinetic stimulus (optokinetic CW) and a counter-clockwise rotating stimulus (optokinetic CCW). A joystick, mounted on a safety bar in front of the subject, could be tilted left or right to produce CCW and CW chair acceleration proportional to the angle of deflection, with a maximum of 30°/s^2^. Turntable and joystick orientation signals were safely stored on a computer hard disk after they have been digitized at 200 Hz for further analysis.

### Experimental Paradigm

**Figure 1:**
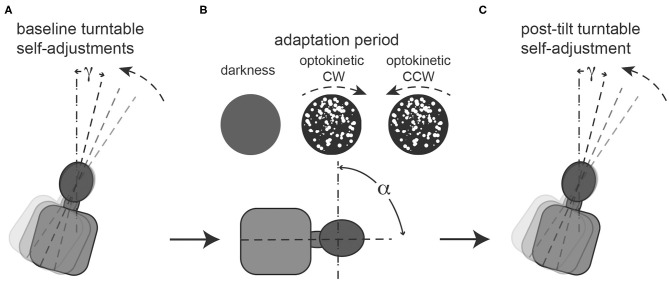
Illustration of the required task at baseline **(A)**, during the 5-min adaptation period **(B)** and immediately afterwards **(C)**. **(A)**: repetitive baseline turntable self-adjustments starting from 90° right-ear down (RED) and 90° left-ear-down (LED) in random order (for illustrative purposes only a single trial starting from 90° RED is show) along the perceived earth-vertical are collected. Angle g represents the deviation of the turntable self-adjustments relative to earth-vertical, thus for perfect self-adjustments along earth-vertical g = 0. **(B)**: during the adaptation period, subjects remain in a static roll-tilted position (referred to as a, set to ±90°, only 90° RED shown for illustrative purposes) either in darkness (“no optokinetic stimulation” condition) or while watching an optokinetic stimulus that is rotating in either clockwise (“optokinetic CW” condition) or counter-clockwise (“optokinetic CCW” condition) direction. No self-adjustments are performed during this period. **(C)**: Single turntable self-adjustment along perceived earth-vertical in darkness (i.e., no optokinetic stimulus is shown) after the adaptation period. Again, g represents the deviation of the turntable self-adjustments relative to earth-vertical.

In all participants, SPV control trials were obtained at the beginning of a single experimental session, followed by the adaptation trials (see Figure 1 for illustration of the experimental paradigm). For all passive turntable movements (e.g., to reach the roll-tilted position for adaptation) we used a constant acceleration and deceleration of ±10°/s^2^. For all subject-guided turntable roll movements the turntable acceleration/deceleration was set to ±30°/s^2^. Importantly, these values were clearly above the detection thresholds of the semicircular canals (19, 20) and for self-motion perception (21). Before data collection, the participants were instructed how to use the joystick and practiced turntable roll movements, thus allowing them to perform the turntable adjustments accurately and precisely.

For the control trials, participants were brought to 90° right-ear down (RED) or 90° left-ear down (LED) position and after being roll-tilted for only 5 s, aligned themselves along perceived earth-vertical in darkness. This was performed 10 times from each starting position, in a pseudo-random order. Afterwards the adaptation trials were recorded, where subjects were roll-tilted 90°, either RED or LED and remained in these roll-tilted positions for 5 min (adaptation period) in each trial. During this period subjects were either kept in darkness (i.e., “optokinetic off” condition) or they were presented a full-field optokinetic stimulus rotating either into the clockwise (“optokinetic CW” condition) or counter-clockwise (“optokinetic CCW” condition) direction. In total, there were six different test conditions (two whole-body roll orientations, three visual stimulus conditions for each whole-body roll orientation) and each condition was recorded three times in every subject. The order of these 18 test trials was random, and after each trial another adaptation period was provided. Between trials (while upright) the lights were turned on briefly.

For both the control trials and the test trials subjects were instructed to move the turntable as quickly and as precisely as possible along the shortest path such that they are in an upright position by use of the joystick. An acoustic signal indicated the start of the subject-guided turntable movement. During these subject-guided adjustments subjects were kept in darkness during all trial conditions. There was no specific time limit, and subjects were required to confirm the completion of adjustments by pushing a button placed next to the joystick.

### Definition of Terms Frequently Used

Clockwise shifts relative to the earth-vertical axis (as seen by the subject) have positive signs, while counter-clockwise shifts have negative signs. We will use the term trial-to-trial variability when referring to the within subject standard deviation (SD). In relation to trial-to-trial variability, the term *precision* reflects the inverse, i.e., the degree of reproducibility. Furthermore, *accuracy* is defined as the magnitude of the mean adjustment error in a given paradigm. For the SPV the direction of rotation was always toward upright and defined by the starting position (either 90°RED or 90°LED).

### Data Analysis

Extracted data from the SPV paradigm was sorted according to the whole-body roll orientation and the different control and test conditions using interactive programs written in Matlab 2017b (The MathWorks, Natick, MA, USA). The chair position when the subject pressed the button to confirm they were finished the adjustment was taken as perceived vertical body position for that trial.

Differences in adjustment errors and variability values for baseline trials and post-adaptation trials were calculated in all subjects. Mean values (±1 SD) were used when pooling individual data points as our data was normally distributed (tested at the level of individual trial conditions using the Jarque-Bera hypothesis test of composite normality, jbtest.m, Matlab 2017b). A generalized linear model (GLM) using SPSS 25 (IBM, Armonk, NY, USA) was applied for all statistical analyses if not specified otherwise. Main effects included the trial condition (*n* = 4; baseline vs. optokinetic off vs. optokinetic CW vs. optokinetic CCW), and the turntable adaptation position (*n* = 2, ±90° roll-tilt). We kept the level of significance at a *p*-value of 0.05, and Fisher's least significant difference (LSD) method was used to correct for multiple comparisons when using the GLM.

The raw data supporting the conclusions of this manuscript will be made available by the authors, without undue reservation, to any qualified researcher.

## Results

Turntable self-adjustments were completed on average after 9.0 ± 2.3 s in all subjects. Statistical analysis yielded no main effect for the starting turntable orientation (df = 1, chi-square = 0.927, *p* = 0.336) and the trial condition (df = 3, chi-square = 7.747, *p* = 0.052) on adjustment time. Furthermore, no significant interactions (df = 3, chi-square = 1.720, *p* = 0.632) were identified.

**Figure 2:**
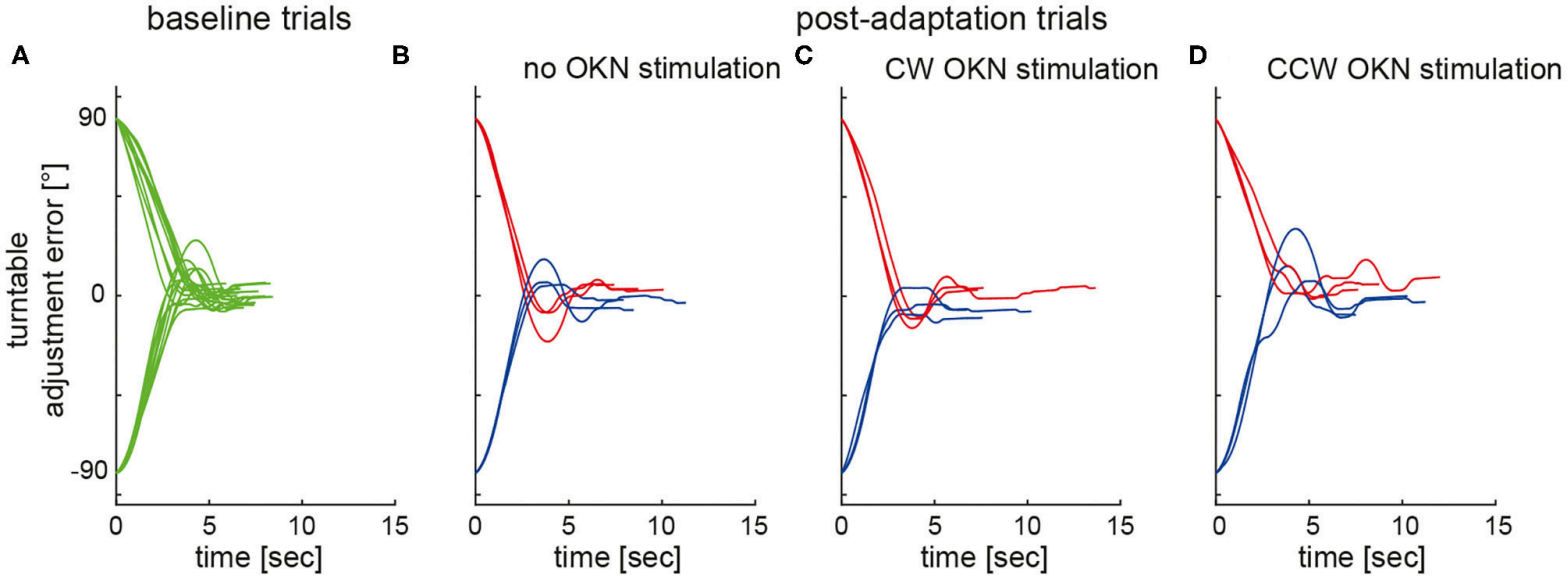
Individual turntable adjustments relative to earth-vertical are plotted against time in a single subject (#10). Whereas, baseline trials **(A)** are shown in green, post-adaptation (test) trials (adaptation period not shown) are plotted in blue (adaptation position: 90° LED) and in red (adaptation position: 90° RED), respectively, for the different test conditions **(B–D)**.

Figure 2 illustrates single SPV adjustments in a typical subject both at baseline (panel A) and after adaptation without (panel B) and with (panels C and D) optokinetic rotatory stimulation, demonstrating a shift of adjustments toward the previous adaptation position in all test conditions.

**Figure 3:**
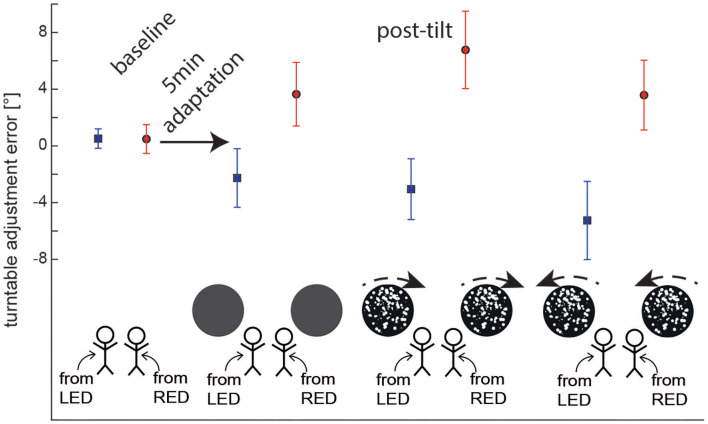
Overall average (±1 SD) turntable adjustment errors are shown for both baseline and post-tilt conditions. For the post-tilt trials the specific adaptation condition (either 90° left-ear down (LED, lines in blue) or 90° right-ear-down (RED, lines in red) and the visual background (no optokinetic stimulus, optokinetic CW, optokinetic CCW) is illustrated. Note that in the post-tilt period all trials were collected in total darkness, i.e., no optokinetic stimulation was present.

For baseline trials, adjustment errors were small, averaging at 0.5 ± 0.7° (mean ± STD) (90°LED adaptation position) and at 0.5 ± 1.0° (90°RED adaptation position), respectively. For the different post-tilt conditions, average offsets ranged between −2.3 ± 2.1 and −5.3 ± 2.8° for 90°LED and between 3.6 ± 2.2 and 6.8 ± 2.7° for 90°RED (see Figure 3 for details).

### Post-tilt Offsets—Effect of Adaptation Position

Statistical analysis (GLM) of post-tilt adjustment errors for the SPV paradigm demonstrated significant main effects both for the condition (df = 3, chi-square = 17.840, *p* < 0.001) and the adaptation position (df = 1, chi-square = 183.216, *p* < 0.001). Furthermore, a significant interaction was found between these two parameters (df = 3, chi-square = 71.751, *p* < 0.001).

Pairwise comparisons indicated significant differences in adjustment errors of the different test trials depending on the adaptation position (90°LED vs. 90°RED) and also in comparison with the control trials (without adaptation). This was true both for those post-adaptation trials without preceding optokinetic stimulation (RED vs. LED, *p* < 0.001) and for those with CW (*p* < 0.001) and CCW (*p* < 0.001) optokinetic stimulation, respectively. In contrast, there was no effect of the starting position (90°LED vs. 90°RED) on adjustment errors in the control trials (*p* = 0.974). Furthermore, adjustment errors were significantly different in post-adaptation (test) trials (with or without optokinetic rotatory stimulation) compared to the baseline trials; this was true both for LED (*p* ≤ 0.002) and RED (*p* ≤ 0.001). In all test conditions, deviations were toward the previous adaptation position, as shown in Figure 3.

### Post-tilt Offsets—Effect of Optokinetic Stimulation

Pairwise comparisons were applied to further assess the observed main effect for the condition (baseline vs. no optokinetic vs. optokinetic CW vs. optokinetic CCW). They demonstrated significant shifts in adjustment errors for trials with optokinetic CW (*p* = 0.034, D = 1.4 ± 0.6°) and optokinetic CCW (*p* = 0.037, D = 1.3 ± 0.6°) stimulation compared to baseline adjustments into the direction of optokinetic stimulation. In contrast, no significant differences were observed comparing test trials without optokinetic stimulation and baseline trials (with both adaptation/starting positions pooled, *p* = 0.763, D = 0.2 ± 0.6°). Comparing the different test trials, SPV adjustments were significantly different between those two conditions with optokinetic stimulation (CW vs. CCW, *p* < 0.001) and between the optokinetic CCW conditions vs. the no optokinetic condition (*p* = 0.017). In contrast, there was no significant difference in offset when comparing the optokinetic CW condition and the no optokinetic condition (*p* = 0.069).

Pairwise comparisons found that the optokinetic stimulation effected the final vertical position only when the optokinetic stimulus moved in the same direction and the adapted title position (that is, CW when RED, and CCW when LED). For SPV adjustments after 5 min adaptation in 90° LED position, adding optokinetic stimulation during the adaptation period resulted in significantly different adjustment errors for optokinetic CCW stimulation (*p* = 0.001), but not for optokinetic CW stimulation (*p* = 0.383) in comparison to darkness (i.e., no optokinetic stimulation) during adaptation. The effect of optokinetic stimulation on turntable adjustment errors was confirmed when directly comparing optokinetic CW and optokinetic CCW conditions (*p* = 0.015).

Likewise, for SPV adjustments after 5 min of adaptation in 90° RED position, adding optokinetic stimulation during the adaptation period resulted in significantly different adjustment errors for optokinetic CW stimulation (*p* = 0.001), but not for optokinetic CCW stimulation (*p* = 0.942) in comparison to the no optokinetic stimulation condition. Again, the effect of optokinetic stimulation on SPV adjustment errors was confirmed when directly comparing optokinetic CW and optokinetic CCW conditions (*p* < 0.001).

### Trial-to-Trial Variability of SPV Adjustments

**Figure 4:**
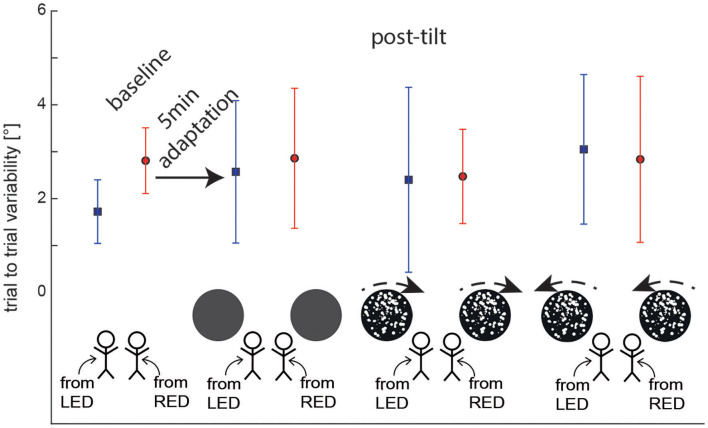
Overall average (± 1 SD) trial-to-trial variability of turntable adjustments are shown for both baseline and post-tilt conditions. For the post-tilt trials the specific adaptation condition (either 90° left-ear down (LED, lines in blue) or 90° right-ear-down (RED, lines in red) and the visual background (no optokinetic stimulus, optokinetic CW, optokinetic CCW) is illustrated. Note that in the post-tilt period all trials were collected in darkness, i.e., no optokinetic stimuli were shown.

Average (±1 SD) trial-to-trial variability for the different baseline and test trials was in the range between 1.7 ± 0.7 and 3.1±1.6° as illustrated in Figure 4. Statistical analysis (again using a GLM) showed no main effect for the starting position (df = 1, chi-square = 1.054, *p* = 0.305) and the trial condition (df = 3, chi-square = 2.985, *p* = 0.394) on trial-to-trial variability. In addition, no significant interactions were noted (df = 3, chi-square = 2.585, *p* = 0.460).

## Discussion

This study was driven by the hypothesis that prolonged static whole-body roll-tilt results in a shift of the internal representation of the direction of gravity (i.e., the “null position”), termed “post-tilt bias” (22), and that visual cues presented during the adaptation period further modulate subsequent self-adjustments. Using a subjective postural vertical (SPV) paradigm, we found turntable self-adjustments to be significantly biased toward the previous adaptation position. For instance, when the subject was previously roll-tilted to the right, the SPV was tilted to the right when subjects were asked to position their body vertically. Presenting an optokinetic rotatory stimulus during the adaptation period resulted in additional offsets in verticality perception, with shifts pointing into the direction of rotation of the visual stimulus. Thus, our data confirms the presence of a post-tilt bias for the SPV paradigm and therefore emphasizes the impact of recent whole-body roll orientation on self-adjustments in the roll plane. These findings also have clinical implications as they point to systematic errors in spatial orientation after prolonged whole-body horizontal orientation, e.g., when getting up in the morning after a good night's sleep, which may contribute to falls and fall-related injuries, especially in the elderly.

### The Effect of Prolonged Whole-Body Static Roll-Tilt on Self-Adjustments Along Perceived Vertical

After 5 min of static whole-body roll-tilt in darkness, we observed average shifts in the SPV of 2.8° (adaptation-positio*n* = 90° LED) and 3.1° (90° RED), respectively. Noteworthy, adjustment errors always deviated toward the previous adaptation position. To our knowledge, such adaptational effects have not been previously described for the SPV. Thus, our data demonstrates that prolonged static whole-body roll-tilt is not only affecting static assessments of verticality perception after returning back upright [by use of e.g., the subjective visual vertical (SVV) or the subjective haptic vertical (SHV)] but is also biasing self-adjustments along perceived direction of gravity. Therefore, our findings further emphasize the impact of the subject's recent orientation relative to gravity on verticality perception.

A post-tilt bias, either toward or (less frequently) away from the previous roll-tilted position, has been previously described for the SVV (2, 7–10) and has been shown to exponentially decay with a median time constant of 71 s (10). More recently, we have demonstrated such a post-tilt bias also for the SHV, i.e., a vision-independent paradigm (11), further supporting the hypothesis that prolonged static roll-tilt results in a mostly paradigm-independent shift of the internal estimate of direction of gravity.

Changes in prior knowledge (or likelihood) in a Bayesian optimal observer model may explain such a shift in the graviceptive null position, as previously discussed by Tarnutzer et al. (10). Specifically, Bayesian optimal observer theory proposes a mechanism where the human brain combines all available sensory cues available in a weighted fashion according to their relative reliabilities and prior likelihood to generate an internal estimate of the direction of gravity (3, 4, 23–26). Thus, in subjects previously being roll-tilted, the prior will be biased toward this roll-tilted position, systematically shifting the resulting posterior probability distribution (4).

Thus, in an experimental setup, shifting prior knowledge has been shown to be a promising approach to study multisensory integration when internally estimating direction of gravity. Shifts in both the SVV, the SHV and the SPV by such adaptational paradigms emphasize the impact of prior knowledge. Noteworthy, besides the subject's roll orientation relative to gravity, also the direction of rotatory optokinetic stimuli and the resulting deviation in mean eye position from normal by an optokinetic nystagmus may bias the prior when assessed by the SPV, as discussed below.

### The Effect of Optokinetic Rotatory Stimuli on Self-Adjustments Along Perceived Vertical

Interestingly, for self-adjustments to perceived vertical (performed in darkness) we observed a significant effect of rotatory optokinetic stimuli presented during the adaptation period. Specifically, SPV adjustments were shifted on average by 1.3–1.4° toward the direction of rotation of the previously presented optokinetic stimulus.

While optokinetic rotatory stimuli have been proven very powerful in biasing verticality perception when using vision-dependent paradigms such as the SVV when presented at the time of the adjustments (15, 27), minor to non-significant shifts only were observed when using other, vision-independent paradigms as the SHV to assess internal estimates of direction of gravity while presenting a rotatory stimulus (16). Noteworthy, an effect of optokinetic stimulation on the SPV, i.e., another, vision-independent paradigm assessing verticality perception, has been described by Dichgans et al. (27). Specifically, asking participants to continuously adjust their whole-body roll orientation to perceived upright while watching a rotatory stimulus, resulted in a shift of 8.5° on average. In contrast, Bisdorff et al. reported no effect of a rotatory optokinetic stimulus (velocity = 60°/s) on the SPV presented during passive whole-body rotation (28).

Furthermore, using the same rotatory optokinetic stimulus during adaptation, we have previously found no significant effect on the subsequent post-tilt bias for both vision-dependent (SVV) and vision-independent (SHV) static paradigms (11). Taking our current findings into consideration, we propose that the effect of prolonged rotatory optokinetic stimuli on verticality perception is paradigm-dependent.

We found an asymmetry in the impact of the optokinetic rotatory stimulus on the SPV. Specifically, significant shifts in turntable self-adjustment errors were noted when the static roll-tilt position and the direction of the rotatory stimulus were into the same (CW or CCW) direction. Thus, shifts were significant when adding CW optokinetic stimulation during whole-body static roll-tilt in 90° RED position (shifting perceived vertical into CW direction as well) and when adding CCW optokinetic stimulation during whole-body static roll-tilt in 90° LED position (shifting perceived vertical into CCW direction as well). In conditions when the prolonged whole-body static roll-tilt and the optokinetic stimulus point in opposite directions, no effect of optokinetic stimulation was observed in comparison to the condition with adaptation performed in darkness. This speaks against a simple additive (or subtractive) effect of these two mechanisms (i.e., the shift in verticality perception by prolonged static whole-body roll-tilt and the shift by prolonged optokinetic rotatory stimulation), but favors a more complex interaction with integration of the visual input only if the tilt direction from upright is in the same direction as the rotating visual stimulus. Thus, our findings propose that for shifting the gravitational null vestibular input is weighted more than concomitant visual input.

Previous studies have demonstrated that the SVV in patients with acute unilateral vestibular loss is strongly tilted toward the side of the lesion, whereas in the same patients the SPV was found to remained veridical, suggesting different weighting of the participating sensory systems for determining the SPV and the SVV (29). These authors concluded that the SPV is derived mainly from somatosensory input, which potentially explains why non-vestibular cues such as optokinetic stimulation had an outlasting effect in our adaptation paradigm when using the SPV but not when using the SVV or the SHV.

In a recently published study, a modulatory effect of dynamic visual stimuli on the SPV was reported (30). Specifically, in this study subjects were first presented a visual stimulus (duratio*n* = 20 s) that was moving downward along the body-longitudinal axis while subjects were roll-tilted 18° to the left side. During the subsequent passive chair rotation to the right side they had to indicate when they felt aligned with earth-vertical. Compared to control trials (without a visual stimulus), test trials with a visual stimulus that was moving downwards with constant acceleration resulted in a shift of subsequent passive SPV adjustments of 0.7° toward the previous roll-tilt position. These findings are consistent with our observation that directed optokinetic stimuli may result in a bias of verticality perception that outlasts the duration of stimulus presentation, consistent with the concept of modulatory effects of prior knowledge in the framework of Bayesian optimal observer theory.

## Limitations

Our study has several limitations, including a moderately large sample size (*n* = 10) and a limited number of trials (*n* = 3) per test condition due to the restriction to a single trial after each adaptation period. We therefore cannot make any conclusions on the decay characteristics of the post-tilt bias for self-adjustments along the perceived direction of gravity. Furthermore, calculations of trial-to-trial variability have to be taken with caution and therefore the reported non-significant differences in variability amongst different trial conditions is a preliminary finding.

## Conclusions

Prolonged static whole-body roll tilt results in a significant “post-tilt” bias of perceived direction of gravity when assessed by the SPV, confirming previous findings from other paradigms including the SVV and the SHV and emphasizing the impact of recent whole-body roll orientation relative to gravity. Such adaptational shifts in verticality estimates may be explained in the context of Bayesian optimal observer theory with a bias of prior knowledge. Furthermore, the significant impact of optokinetic rotatory stimuli on subsequent self-adjustments along perceived vertical, whereas such an effect was not found for the SVV and the SHV previously, is potentially explained by differences in weighting of the sensory input available when centrally integrated. Our findings also have clinical implications, as the observed post-tilt bias may contribute to postural instability when standing up in the morning, increasing the risk for falls and fall-related injuries in patients with preexisting balance disorders.

## Data Availability Statement

The raw data supporting the conclusions of this article will be made available by the authors, without undue reservation.

## Ethics Statement

The studies involving human participants were reviewed and approved by Cantonal Ethics Committee Zurich. The patients/participants provided their written informed consent to participate in this study.

## Author Contributions

AW: collection, analysis and interpretation of data, and drafting and revising the article critically for important intellectual content. CB: assisted in the design of the experiments, analysis and interpretation of data, and revising the article critically for important intellectual content. DS: assisted in the design of the experiments and revising the article critically for important intellectual content. AT: conception and design of the experiments, collection, analysis and interpretation of data, and revising the article critically for important intellectual content. All authors: have approved the final version of the manuscript, all persons designated as authors qualify for authorship, and all those who qualify for authorship are listed.

## Conflict of Interest

The authors declare that the research was conducted in the absence of any commercial or financial relationships that could be construed as a potential conflict of interest.
